# Fitness of calves born from *in vitro*-produced fresh and cryopreserved embryos

**DOI:** 10.3389/fvets.2022.1006995

**Published:** 2022-11-24

**Authors:** Enrique Gómez, Antonio Murillo, Susana Carrocera, Juan José Pérez-Jánez, Jose Luis Benedito, David Martín-González, Isabel Gimeno

**Affiliations:** ^1^Servicio Regional de Investigación y Desarrollo Agroalimentario (SERIDA), Centro de Biotecnología Animal, Gijón, Spain; ^2^Cooperativa de Agricultores y Usuarios de Gijón, Gijón, Spain; ^3^Department of Animal Pathology, Veterinary School, University of Santiago de Compostela, Lugo, Spain

**Keywords:** bovine, embryo-freezing, calves, embryo-vitrification, creatinine, acid-base

## Abstract

In cattle, vitrified/warmed (V/W) and frozen/thawed (F/T), *in vitro*-produced (IVP) embryos, differ in their physiology and survival from fresh embryos. In this study, we analyzed the effects of embryo cryopreservation techniques on the offspring. IVP embryos cultured with albumin and with or without 0.1% serum until Day 6, and thereafter in single culture without protein, were transferred to recipients on Day 7 as F/T, V/W, or fresh, resulting in *N* = 24, 14, and 13 calves, respectively. Calves were clinically examined at birth, and blood was analyzed before and after colostrum intake (Day 0), and subsequently on Day 15 and Day 30. On Day 0, calves from V/W and F/T embryos showed increased creatinine and capillary refill time (CRT) and reduced heartbeats. Calves from F/T embryos showed lower PCO_2_, hemoglobin, and packed cell volume than calves from V/W embryos while V/W embryos led to calves with increased Na^+^ levels. Colostrum effects did not differ between calves from fresh and cryopreserved embryos, indicating similar adaptive ability among calves. However, PCO_2_ did not decrease in calves from V/W embryos after colostrum intake. Serum in culture led to calves with affected (*P* < 0.05) temperature, CRT, HCO${}_{3}^{-}$, base excess (BE), TCO_2_, creatinine, urea, and anion gap. On Day 15, the effects of embryo cryopreservation disappeared among calves. In contrast, Day 30 values were influenced by diarrhea appearance, mainly in calves from V/W embryos (i.e., lower values of TCO_2_, HCO${}_{3}^{-}$, and BE; and increased glucose, anion gap, and lactate), although with no more clinical compromise than calves from fresh and F/T embryos. Diarrhea affected PCO_2_ and Na^+^ in all groups. Embryo cryopreservation, and/or culture, yield metabolically different calves, including effects on protein and acid–base metabolism.

## Introduction

*In vitro* assisted reproductive technologies are sustained by cryopreservation of gametes and embryos. Cryopreservation suppresses the necessity to have recipients available at the time of embryo production, thus facilitating worldwide exchanges of genetic material. In cattle, cryopreservation of *in vitro*-produced (IVP) embryos for embryo transfer (ET) takes place normally after 7 ± 1 days of culture. Embryo cryopreservation (covering slow freezing and vitrification) induces damage such as morphological alteration (1), DNA fragmentation (2), decreased cell numbers and increased apoptotic cell ratio (3–5), abnormal gene expression (6–8) and histone modifications (9), and changes in embryo metabolism (10). Adaptation of embryos to divergent conditions may trigger changes in post-natal phenotypes that may persist until adulthood, as observed in rats (11, 12). In cattle and other mammalian species, *in vitro* embryo production technologies lead to offspring phenotypes deviating from those naturally conceived or resulting from the transfer of *in vivo* collected embryos (13–15). An extreme example occurs among calves born from somatic cell nuclear transfer (SCNT) (16–18).

Generally, IVP cryopreserved embryos are less able to reach term pregnancy upon transfer than their fresh counterparts and *in vivo*-produced embryos (4, 19). Cryopreservation reduces embryo survival rates and cell numbers *in vitro* (3, 4, 20, 21) and causes a series of functional and structural damage in embryonic cells [reviewed by Mogas (22)]. At the transcriptomics level, vitrification itself alters the expression of genes relevant for development in IVP embryos surviving cryopreservation (3, 23–25). Thus, IVP embryos show altered cell differentiation, lipid metabolism, and cell adhesion, with the intensity of such changes being higher in vitrified than in frozen embryos (6). Moreover, embryonic genes associated with organogenesis, immune response, and regulation of cognitive functions are altered after vitrification, compared to fresh embryos (25). Extensive transcriptomic changes remain within vitrified/warmed embryos exposed to the uterus and collected on Day 14, which show alteration in cell proliferation, cellular stress response, mitochondrial dysfunction, control of translation initiation, and DNA repair (23).

However, *in vitro* experiments often do not accurately represent embryonic survival *in vivo*, and, while *in vitro* quality parameters are sometimes superior in vitrified/warmed (V/W) over frozen/thawed (F/T) embryos, such differences are not observed in pregnancy rates (4, 26). Indeed, ET reports with large numbers of V/W and F/T IVP embryos indicate that both cryopreservation systems perform equally in pregnancies reaching term, sometimes with rates close to fresh IVP embryos (4, 13, 19, 27–30). Such studies focused on pregnancy and/or birth rates, sometimes combined with calf morphometry and survival; nevertheless, how V/W and F/T affect calf phenotypes and basic hematological parameters is presently unknown.

A proportion of calves born from IVP embryos are prone to show larger birth weight (BW) and perinatal mortality (14), under a phenotype known as large offspring syndrome (LOS) or abnormal offspring syndrome (AOS) (31–33) that courses with placental overgrowth (34, 35) and compromised vascular development (18, 36, 37). However, subtle alterations may remain within animals derived from IVF procedures (38), even without obvious phenotypic abnormality (33, 38); thus, such animals are normally incorporated into the productive process in farms. Clinical studies detected differences between calves born from IVP embryos compared to calves born from artificial insemination (AI) (39, 40), and between these and clones (41). Furthermore, male IVP calves show activation of the hypothalamus–pituitary–gonadal axis earlier than *in vivo* developed calves produced by multi-ovulation and embryo transfer (MOET) (38).

Neonates experience a striking metabolic challenge at birth. The placental nutrition ceases abruptly and is replaced by a cyclic enteral food supply to conserve homeostasis (42). Such a change requires progressive adaptation to adulthood with adrenal, pancreatic, and thyroid hormones driving from fetal setpoints to anabolic oxidative metabolism, energy storage, and tissue accretion (43). The welfare of calves has been often analyzed by measuring biochemical and hematological parameters (39–41, 44–46). Such studies are crucial because of their contribution to defining normalcy and alteration intervals. In contrast, limited knowledge of the effects of cryopreservation on calf fitness was documented.

In the present study, we hypothesized that cryopreservation, as one of the hallmarks that trigger alterations and reduce the viability of IVP embryos, may underlie effects on calf fitness that cannot be obvious in offspring. For this purpose, we used embryo cryopreservation procedures that yielded gestation length (GL), BW, and daily gain weight of the fetus that did not differ between fresh, F/T, and V/W calves (4). This way, the absence of labor difficulties derived from heavier calves and/or extended gestation may facilitate the evaluation of the effects of cryopreservation as is or as a specific technique in itself (i.e., F/T or V/W).

## Materials and methods

The study was conducted following the guidelines of the Declaration of Helsinki and approved by the Animal Research Ethics Committees of SERIDA and the University of Oviedo (PROAE 33/2020; Resolución de 13 de Noviembre de la Consejería de Medio Rural y Recursos Naturales), in accordance with European Community Directive 86/609/EC.

All reagents were purchased from SIGMA (Madrid, Spain) unless otherwise stated.

Calves used in this study were born in a 3-year period (May 2017-July 2020) from IVP embryos transferred to recipients following procedures that did not induce differences between fresh, frozen/thawed (F/T), and vitrified/warmed (V/W) embryos in calf BW and GL (4) An update of animals born in our herd confirmed no differences in BW average, although 5/35 F/T and 8/33 V/W cases showed BW>50 kg, compared with 1/26 in fresh embryos (3, 3, and 1 of them, respectively, used in this study; unpublished data). However, only two calves born from V/W and fresh embryos and pregnancies >290 days showed ≥60 kg at birth (4). For these experimental purposes, the calves available for analysis at Day 0 were *N* = 13 (fresh), *N* = 14 (V/W), and *N* = 24 (F/T) (refer to Table 1, for details). The readers are referred to our articles (4, 24, 47) for a complete description of oocyte collection from slaughterhouse ovaries, *in vitro* maturation (IVM) and *in vitro* fertilization (IVF), which was performed with frozen/thawed semen from single seven individual bulls [three Holstein and four Asturiana de los Valles (AV)]. Pregnancy rates from each procedure were also described (4, 24). An update of embryo transfers performed in our experimental herd, pregnancy, pregnancy loss, birth rates, and calving ease for fresh, F/T, and V/W embryos cultured with or without serum is shown in Table 2.

**Table 1:** Calves sampled according to their embryonic origin (i.e., cryopreservation) and timing before or after colostrum intake, and calves deceased during the sampling period (30 days from birth).

	**Colostrum intake**				**Diarrhea**		**Non-Diarrhea**	
**Embryo**	**Before**	**After**	**Male**	**Female**	**Treated**	**Untreated**	**Dead**	**Dead**
Fresh	12	13	5	8	2	1	0	0
Vitrified	10	14	7	7	2	2	0	1
Frozen	19	23	17	7	5	4	2	2

Diarrhea cases before Day-15 (*N* = 3) and after Day-15 (*N* = 13; mostly affecting Day-30 measurements by diarrhea/or its treatment).

Deaths: 1 vitrified (non-diarrhea: Day-2); 2 frozen (non-diarrhea: 1 Day-2 and 1 Day-7; diarrhea: 1 Day-10 and 1 Day-12).

Total calves: 24 Frozen; 13 Fresh; 14 Vitrified.

**Table 2:** Update of Day 40 and Day 62 pregnancy rates, pregnancy loss rates, birth rates, and calving ease after transfer of Day 7 and Day 8 vitrified, frozen, and fresh embryos cultured from Day 0 to Day 6 in groups with either BSA (0.6%) or FCS (0.1%) + BSA (0.6%), and subsequently in individual culture without protein supplements (0.5 mg/ml PVA) from Day 6 to Day 7 in the experimental herd.

				**Pregnancy rates (%)**		**Pregnancy loss >Day 40**		**Calving ease (** ***N*** **) %**			
**Embryo**	**Culture**	**Day**	***N***	**Day 40**	**Day 62**		**Birth rates (%)**	**1 and 2**	**3 and 4**	**5**	**Calves analyzed**
Fresh	BSA	7	32	(22/32) 68.7	(18/32) 56.2	21.8 (7)	(15/32) 46.9	(15) 100	-	-	7
	FCS+BSA	7	17	(11/17) 64.7	(10/17) 58.6	27.3 (3)	^*^(8/16) 50.0	(7) 87.5	-	(1) 12.5	5
Vitrified	BSA	7	50	(32/50) 64.0	(31/50) 62.0	25.0 (8)	(24/44) 54.5	(21) 87.5	(3) 12.5	-	10
	BSA	8	2	(1/2) 50.0	(1/2) 50.0	-	(1/2) 50.0	(1) 100	-	-	-
	FCS+BSA	7	10	(5/10) 50.0	(5/10) 50.0	20.0 (1)	^*^(4/9) 44.4	(4) 100	-	-	4
Frozen	BSA	7	65	(36/65) 55.4	(34/65) 52.3	13.8 (9)	(27/59) 45.8	(23) 85.2	(4) 14.8	-	18
	BSA	8	17	(5/17) 29.4	(5/17) 29.4	40.0 (2)	(4/17) 26.7	(4) 100	-	-	2
	FCS+BSA	7	17	(9/17) 52.9	(8/17) 47.0	29.4 (5)	(4/17) 26.7	(3) 75	(1) 25	-	4

^*^Two recipients dead after fresh ET (1 open and 1 pregnant after Day 62). Birth rates are unmatched with pregnancy rates reflecting that some pregnancies are ongoing. Of the total of calves born at term, five died 24 h after birth, four corresponding to a transfer of a vitrified embryo cultured with BSA, and one to a fresh embryo cultured with BSA. Calving ease, 1 (no assistance required); 2 (soft traction without manipulation); 3 (hard traction); 4 (manipulation and traction); and 5 (cesarean section).

Distribution of samples used per bull, Bull A, *N* = 12; Bull B, *N* = 9; Bull C, *N* = 10; Bull D, *N* = 4; Bull E, *N* = 7; Bull F, *N* = 7; Bull G, *N* = 2.

The calves analyzed in the present study are detailed.

### *In vitro* embryo production

Extended *in vitro* culture (IVC) procedures were described (47). Briefly, after IVF, presumed zygotes were loaded into the embryo culture medium (CM) which consisted of modified synthetic oviduct fluid (mSOF) with MEM non-essential amino acids (M7145), BME amino acids (B6766), citrate (0.1 μg/ml), myo-inositol (0.5 μg/ml), and Bovine serum albumin (BSA) (A3311) (6 mg/ml) with or without 0.1% (v/v) FCS (SIGMA F4135), under mineral oil at 38.7°C, 5% CO_2_, 5% O_2_, 90% N_2_, and saturated humidity. Embryos were cultured in groups until Day 6. On Day 6 (143 h after IVF onset) morulae, early blastocysts and blastocysts were cultured individually in 12-μl protein-free mSOF with polyvinyl-alcohol (P8136) under mineral oil for 24 h. Thereafter, Day 7 embryos at the expanding blastocyst stage (ExB) and fully expanded blastocysts (FEB) were transferred fresh or cryopreserved (F/T or V/W) to recipients synchronized on cycle Day 7. As an exception, one calf was derived from one Day 7 embryo which was re-cultured for 24 h with new protein-free CM and transferred as an F/T Day 8 embryo.

### Embryo freezing and thawing

Freezing and thawing followed a described procedure (4). Only ExB and FEB were collected from single culture drops and washed three times in phosphate buffered saline (PBS) + 4-g/L BSA and loaded in a freezing medium containing PBS (P4417), 1.5-M EG, and 20% CRYO3 (5617, Stem Alpha, France) for 10 min. Embryos were loaded into a French straw between two columns with PBS + 0.75-M EG + 20% CRYO3, and two further columns PBS + 0.75-M EG + 20% CRYO3 separated by air. The straw was sealed with a plug and loaded into a programmable freezer (Crysalis, Cryocontroller PTC-9500) at −6°C for 2 min and seeded once with supercooled forceps. Straws remained for 8 min at −6°C and were subsequently dehydrated at a −0.5°C/min rate up to reach −35°C. Finally, the straws were stored in LN_2_ until used for ET. Thawing was performed by holding the straws for 10 s on air and 30 s in at 35°C (water bath) and drying with 70% ethanol. Each thawed straw containing a single embryo was mounted in an ET catheter and non-surgically transferred to recipients deeply in the uterine horn ipsilateral to the corpus luteum of synchronized recipients.

### Embryo vitrification

Vitrification procedures have been previously described (48). Only ExB and FEB blastocysts were vitrified in two steps with fibreplugs (CryoLogic Vitrification Method; CVM), working on a heated surface (41°C) in a warm room (25°C). Embryos were handled in a basic vitrification medium (VM: TCM 199-HEPES + 20% (v/v) FCS). Each blastocyst was exposed to VM with 7.5% ethylene-glycol (EG, 102466-M), 7.5% dimethyl sulfoxide (DMSO) (D2650, vitrification solution-1) for 3 min, and then moved into a drop containing VM with 16.5% EG, 16.5% DMSO and 0.5-M sucrose (vitrification solution-2; VS2). The time spent by the embryos in VS2 (including loading) did not exceed 25 s. Sample vitrification was induced by touching the surface of a supercooled block placed in LN_2_ with a hook. Fibreplugs with the vitrified embryos were stored in closed straws in LN_2_ until warming previous to ET. Embryos were warmed in single-step by directly immersing the fibreplug end in 800 μl of 0.25 M sucrose in VM, where the embryo was kept for 5 min and subsequently washed twice in VM and twice in mSOF containing 6-mg/ml BSA and 10% FCS before preparing for ET.

### Recipient management, embryo transfer, and pregnancy diagnosis

All recipients were managed, fed, transferred, and housed during gestation; calving; and perinatal period (30 days) in the experimental herd. Such procedures have been described in depth (49, 50) and performed to minimize environmental differences. Embryos were transferred to AV, Holstein, and crossbred recipients synchronized in estrus with a progestagen-releasing device (PRID Alpha; CEVA, Barcelona, Spain), loaded intravaginally for 8–11 days, and removed 48 h after prostaglandin F2α analog (Dynolitic, Pfizer, Madrid, Spain) injection. Estrus appearance was observed by experienced caregivers 2–3 times per day and/or monitored with an automated sensor system (Heatphone, Medria, Humeco, Huesca, Spain). In the absence of clear estrous signs, progesterone concentrations were measured to select recipients, with P4 fold change Day 7/Day 0 >8 and Day 7 P4 values >3.5 ng/ml. An enzyme-linked immunosorbent assay (ELISA) test (EIA-1561, DRG Diagnostics, USA) was used for progesterone measurement. Before ET, all recipients were clinically explored by ultrasonographic scanning for detection of a corpus luteum in one ovary and transferred at a fixed time. ETs were performed non-surgically under epidural anesthesia. Fresh embryos were washed twice in Embryo Holding Media (019449, IMV Technologies) and mounted in straw in the same medium. Vitrified embryos were warmed, examined in their morphology, and mounted as fresh embryos for transfer. Frozen/thawed embryos were directly transferred in straw and not examined. Pregnancy was diagnosed on Day 40 and Day 62 by ultrasonography and birth rates were monitored.

### Rationale

We planned a study based on sample variability to reach experimental randomness. The variability in the experimental conditions and individual bulls is intended to add degrees of freedom to our study and avoid linkage between any particular condition (i.e., a specific embryo culture, a single bull, etc.) and the parameters we analyzed among calves from fresh, F/T, and V/W embryos. Gestation was allowed to end naturally in the experimental herd without calving induction. Calving and birth time were monitored with an intravaginal sensor (Vel'Phone, Medria, Humeco, Huesca, Spain). After birth, mothers were adjusted in milk production by feeding 3 kgs concentrate/day, given by automated dispenser; supervised manual milking was performed when necessary to reduce possible excess of milk production in dairy mothers. Calves were kept with mothers in free stalls and suckled colostrum and milk ad libitum in order to satisfy milk amounts >20% of calf body weight (51). Mothers and calves were held in the same shared environment until calves were aged 1 month. The health status of calves was assessed daily by experienced caregivers who monitored behavior changes, suckling and appetite, fatigue, diarrhea (presence/ absence), cough, eye, and/or nasal discharges. At birth (Day 0), blood samples were collected before colostrum intake and 1–4 h after colostrum intake (52). Hematological and biochemical analysis were performed on blood samples taken subsequently at fixed times (10 a.m. on Days 15 ± 1 and 30 ± 1 of life), together with clinical examination. Blood was collected in vacuum tubes (containing lithium heparin) from the jugular vein, shaking 10 times, and directly analyzed in a Vetscan i-STAT One analyzer (Scil Animal Care, Madrid, Spain; CG4+ and CHEM8+ modules). The analyzer was loaded in a room in the experimental farm kept at 25°C, and blood tubes were collected independently to minimize time spent until analysis. Parameters measured in calves and taken into account in embryos for analysis are shown in Table 3. Parameters measured by Vetscan i-STAT One were previously subjected to a time-course validation to check consistency and, eventually, apply corrections (which were not necessary as all measures were performed in blood collected in time). Within the measured parameters, acid–base status was estimated through the followings: partial pressure of CO_2_ (PCO_2_), which represents the respiration fraction of acid–base balance (i.e., Cellular production of CO_2_ and ventilatory removal of CO_2_); HCO${}_{3}^{-}$, which is the metabolic component of acid–base balance; total CO_2_ (TCO_2_) is a measure of carbon dioxide in several states: CO_2_ in physical solution or loosely bound to proteins, HCO${}_{3}^{-}$ or CO_3_ ions and carbonic acid (H_2_CO_3_); anion gap (AG), which detects organic acidosis by measuring differences between the cations Na^+^ and K^+^ and the anions Cl^−^ and HCO${}_{3}^{-}$; PO_2_, i.e., partial pressure of oxygen dissolved in blood; oxygen saturation (sO_2_), which represents the total amount of hemoglobin (Hb) able to bind oxygen—oxyhemoglobin plus deoxyhemoglobin; base excess (BE), which is the non-respiratory component of pH; and pH.

**Table 3:** Variables controlled in embryos and parameters measured in calves on Days 0, 15, and 30 after birth.

**Parameter**	**Details**
Recipient Breed	AV / Holstein / Crossbred
Mother weight at birth	Kg
Calving easy	AU: 1-5 (no intervention to Caesaran section)
Gestation length	Days
Calf breed	AV / Holstein / Crossbred
Calf sex	Male / Female
Colostrum intake	Yes / No
Suckling reflex	Yes / No
Capillary refill time	Seconds: 1-5 (time to recover color after gum pressure)
Rectal temperature	°C
Conjunctival appearance	AU: 1-Anemic; 2-Pale; 3-congestive; 4 icteric
Nasal mucosa appearance	AU: 1-No discharge; 2-Discharge; 3-Colored discharge
Ganglion size	Palpation: 0-not-increased; 1-increased
Ganglion pain	Digital pressure: No / Yes
Calf weight	Kg
Body Size	Cm
Chest perimeter	Cm
Heartbeats	Beats / min (auscultation)
Respiration	Breathings /min (auscultation)
pH	Vetscan i-STAT One (CG4+)
CO_2_ partial pressure (PCO_2_)	mm Hg (Vetscan i-STAT One; CG4+)
O_2_ partial pressure (PO_2_)	mm Hg (Vetscan i-STAT One; CG4+)
Base excess (BE)	mmol/L (Vetscan i-STAT One; CG4+)
HCO${}_{3}^{-}$	mmol/L (Vetscan i-STAT One; CG4+)
Total CO_2_ (TCO_2_)	mmol/L (Vetscan i-STAT One; CG4+)
O_2_ saturation (sO_2_)	% (Vetscan i-STAT One; CG4+)
Lactic acid	mmol/L (Vetscan i-STAT One; CG4+)
Na^+^	mmol/L (Vetscan i-STAT One; Chem8+)
K^+^	mmol/L (Vetscan i-STAT One; Chem8+)
Cl^−^	mmol/L (Vetscan i-STAT One; Chem8+)
Ca^2+^	mmol/L (Vetscan i-STAT One; Chem8+)
Glucose	mg/dL (Vetscan i-STAT One; Chem8+)
Urea	mg/dL (Vetscan i-STAT One; Chem8+)
Creatinine	mg/dL (Vetscan i-STAT One; Chem8+)
Hematocrit	% PCV^(a)^ (Vetscan i-STAT One; Chem8+)
Hemoglobin	g/dL (Vetscan i-STAT One; Chem8+)
Anion gap	mmol/L (Vetscan i-STAT One; Chem8+)

^(a)^ Packed cell volume. AV: Asturiana de los Valles. AU, Arbitrary units.

### Statistical analysis

Statistical analysis was performed with SAS/STAT package (Version 9.2; SAS Institute, Inc.) using GLM models. The biochemical and hematological parameters analyzed were first submitted to a time-course validation test for consistency to obtain appropriate time intervals for chemometric analysis. Subsequently, for their study, data were divided into two sets (Day 0, with and without colostrum intake; and a time-course Day 0, Day 15, and Day 30 analysis, excluding Day 0 samples obtained prior to colostrum intake). This second analysis included the effects of diarrhea, which appeared at specific time points in some calves (refer to the footnote in Table 1). Major effects alone and/or combined with pre-planned interactions were studied in both datasets. First, the effect of cryopreservation systems on calf fitness on Day 0 was analyzed in combination with or without colostrum intake. The following major effects were considered and weighed in the GLM model: the origin of the calf based on embryo cryopreservation (fresh, frozen, and vitrified); colostrum intake; embryo culture medium prior to Day 6 (i.e., with BSA or BSA+FCS); calf sex; calf breed (Holstein, AV, and crossbred); and individual bull and recipient breed (random effects). Second, the time-course effects of cryopreservation on the original embryos were analyzed in their interaction with blood sampling times on Day 0 (after colostrum intake), Day 15, and Day 30. The major effects included in the models were as above, except those made of colostrum intake and included diarrhea as a random effect. Parameters that did not show significant interactions between cryopreservation and sampling day were analyzed singly in the time course (i.e., Day 0, Day 15, and Day 30). Data were expressed as LSmeans ± SEM. The *P*-values of < 0.05 were considered significant for variable values analyzed within each model. Subsequently, the predicted least square mean difference value (PDIFF) was calculated as a *post-hoc* test to identify significant differences (*P* < 0.05) between least square means.

## Results

### Analytical validation

The stability of hematological and biochemical parameters was tested in samples through a time-course experiment (Supplementary Tables S1A, B). Within the CG4+ analytical module, no parameter showed significant deviations in measured concentrations before an average reading time of 10.75 min (range: 10–11 min). Only lactate showed significant (*P* < 0.01) differences with the precedent times at a reading time of 14.75 min (range: 14–16 min). Parameters analyzed by the CHEM8+ module did not differ at any analytical time (top time: 13.2 min, range: 12–14 min). In accordance with these validation studies, all samples were analyzed and read < 6 min after blood collection.

### Studies on Day 0

Day 0 samples analyzed were *N* = 91 (*N* = 41 before and *N* = 50 after colostrum intake), taken from *N* = 51 calves (frozen: 24; fresh: 13; vitrified: 14) (refer to Table 1). Within such a dataset, BW was affected by embryo culture and sex but not by embryo cryopreservation; chest perimeter by embryo culture, and calf size by sex (Table 4). In the main effects shown in Table 4, the bull (individual random effect) had the largest influence, with 17 parameters being affected, most of them with strong significance (*P* < 0.01). Colostrum intake affected eight parameters, while cryopreservation and embryo culture influenced 7 and 10 values measured in calves, respectively. Recipient breed affected four parameters, calf sex seven parameters, and calf breed had no effect. Lactate, respiration rates, PO_2_, and sO_2_ were independent of all variables analyzed.

**Table 4:** Main effects from clinical signs and blood parameters in calves born from fresh, vitrified, and frozen embryos measured on Day 0 prior to and after colostrum intake.

			**Embryo**	**Calf**		**Recipient**	
**Parameter**	**Colostrum**	**Cryo^(a)^**	**Culture**	**Sex**	**Breed**	**breed**	**Bull**
CRT		< 0.001	0.010				0.010
Birth weight			< 0.001	0.003			0.005
Chest perimeter			< 0.001				0.001
Size				0.012			0.001
Temperature	0.010		< 0.001				
Conjunctival	0.047					0.016	
Nasal flux						< 0.001	
Heartbeats	**0.051**	0.034					< 0.001
Respiration							
pH	< 0.001						0.002
PCO_2_	< 0.001	0.008					0.015
Base excess	0.005		0.033				0.004
HCO${}_{3}^{-}$	0.037		0.011				
TCO_2_			0.012				0.015
Lactate							
Na^+^		0.040		0.012		< 0.001	< 0.001
K^+^							0.027
Cl^−^				0.003		< 0.001	
Ca^2+^	0.027						< 0.001
Glucose							0.010
Urea			0.023				0.001
Creatinine		0.004	0.030	0.004			
PCV		0.046		0.005			< 0.001
Hemoglobin		0.044		0.005			< 0.001
Anion gap			0.027				< 0.001
PO_2_							
sO_2_							

^(a)^Cryo: embryo fresh or cryopreserved (vitrified or frozen). CRT, capillary refill time. Tendency values (0.05>*P* < 0.06) are shown in bold.

**Figure 1:**
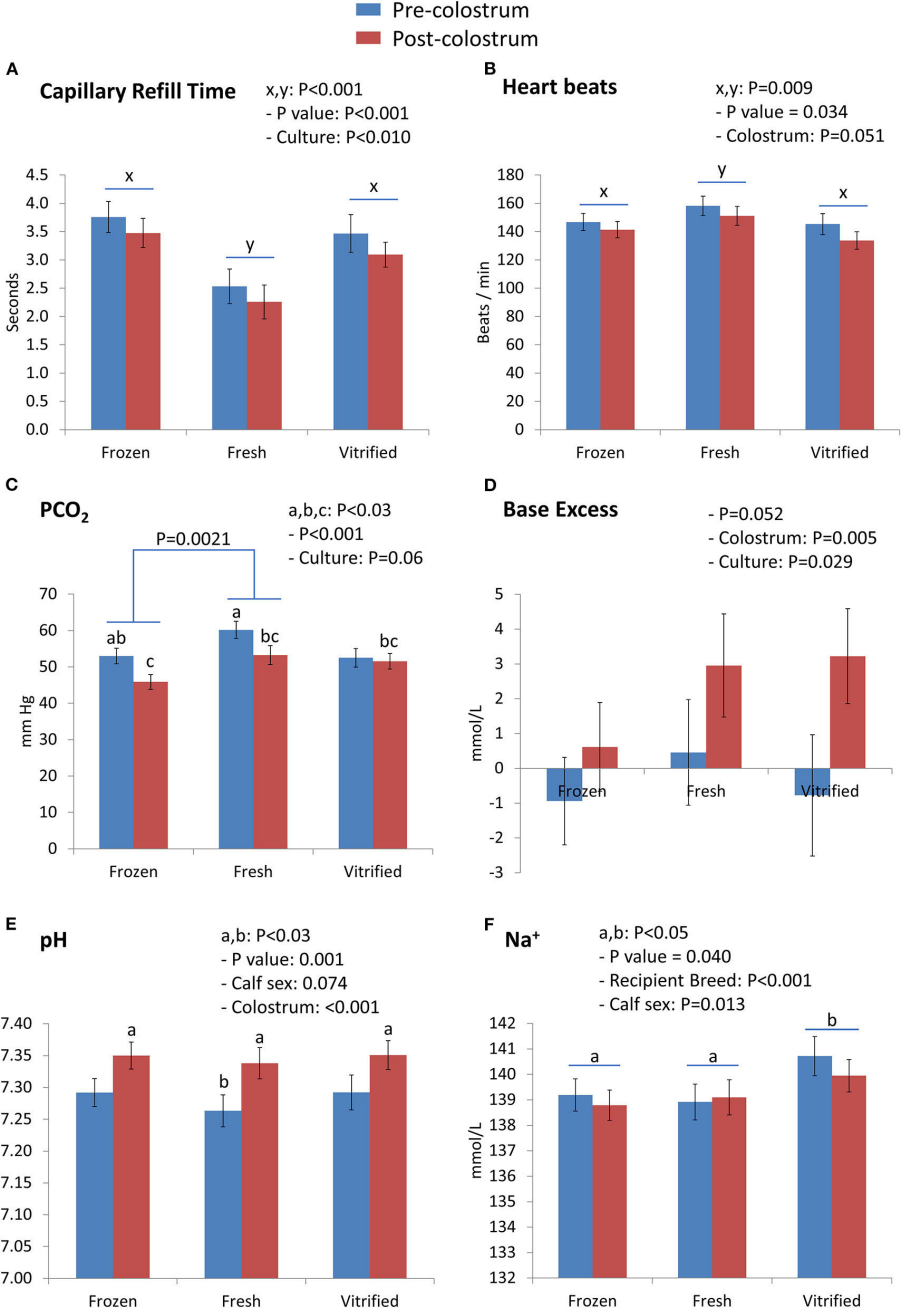
Clinical traits [capillary refill time **(A)** and heartbeats **(B)**] and acid–base equilibrium and blood gases parameters [PCO_2_ **(C)**, base excess **(D)**, pH **(E)**, and Na^+^ **(F)**] differed on Day 0 between groups of calves (from frozen/thawed, fresh, and vitrified/warmed embryos). Values are LSM ± SEM. Samples were *N* = 42 pre-colostrum and *N* = 50 post-colostrum, corresponding to *N* = 24 for the frozen group, *N* = 13 for the fresh group, and *N* = 14 for the vitrified group of calves. Underlined superscripts indicate the cryopreservation effect, and not-underlined superscripts indicate the interaction between the cryopreservation system and colostrum intake.

**Figure 2:**
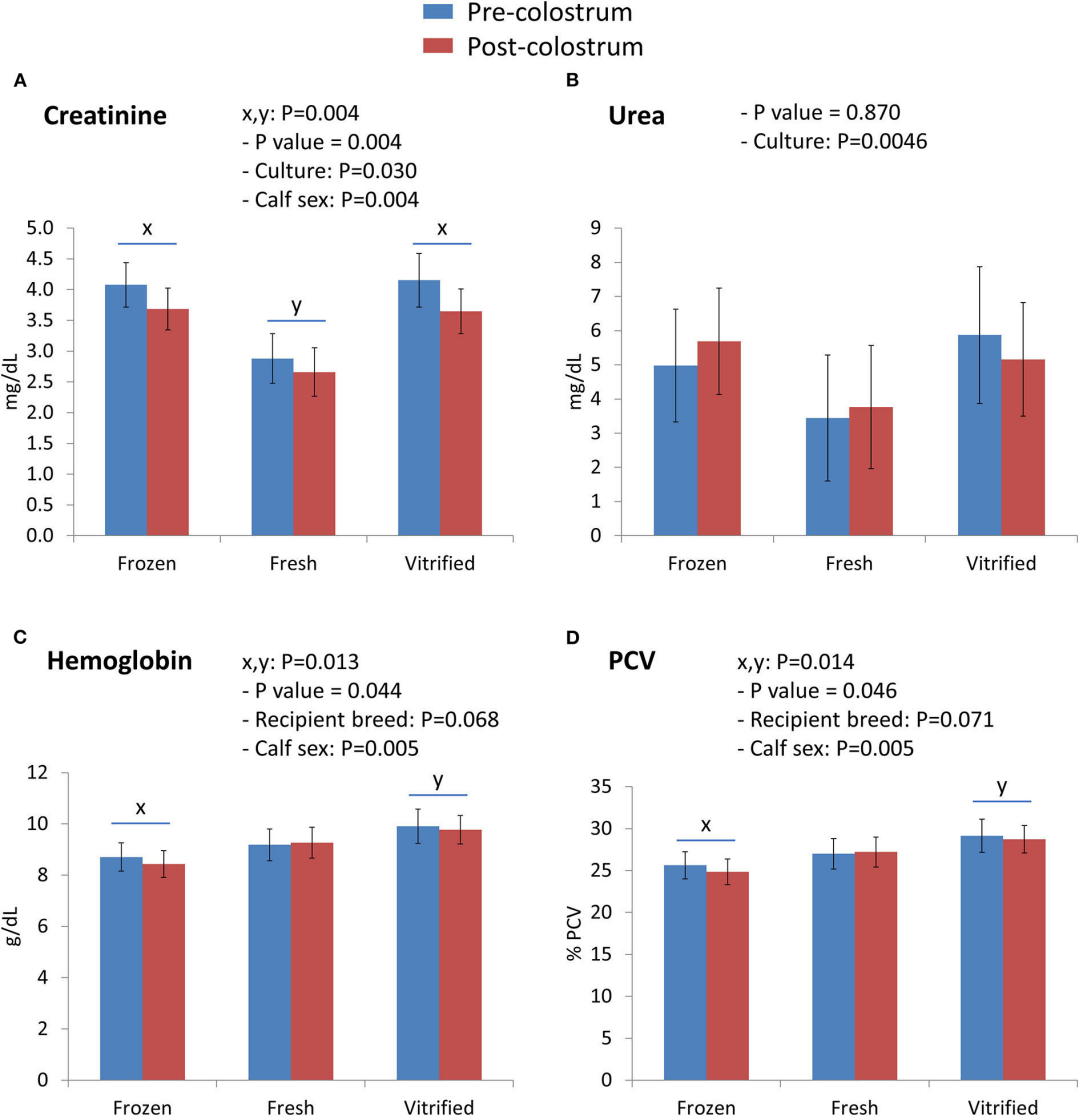
Metabolite parameters [creatinine **(A)** and urea **(B)**] and hematological variables [hemoglobin **(C)** and packed cell volume **(D)**] differed on Day 0 between calves born from frozen/thawed, fresh, and vitrified/warmed embryos. Data are expressed as LSM ± SEM. Underlined superscripts (x,y) indicate the cryopreservation effect.

Interactions between embryo cryopreservation systems and colostrum on Day 0 are shown in Figures 1, 2. Notably, relevant effects of both embryo cryopreservation systems on clinical traits in calves (Figure 1) were observed for capillary refill time (CRT) and differed significantly (*P* < 0.001) from the value in calves of fresh embryos (calves of F/T: 3.613 ± 0.232 s and calves of V/W embryos: 3.235 ± 0.247 s; vs. calves of fresh embryos: 2.394 ± 0.252 s) (Figure 1A) and heartbeat rate (calves from fresh: 154.6 ± 5.6 vs. F/T: 143.9 ± 5.2 and from V/W embryos: 138.8 ± 5.5; *P* < 0.034) (Figure 1B). Within acid–base equilibrium and blood gases (Figure 1), differences between cryopreservation systems were also recorded for PCO_2_ (*P* < 0.01) (Figure 1C), between calves derived from F/T embryos (49.55 ± 1.86 mm Hg) vs. calves from fresh embryos (56.80 ± 2.02) but not within calves from V/W embryos (52.71 ± 1.97); at the same time, colostrum intake did not reduce PCO_2_ in calves of V/W embryos, contrary to in calves from F/T and fresh embryos. Base excess (Figure 1D) and pH (Figure 1E) were not affected by cryopreservation but they showed a significant restorative effect of colostrum (*P* = 0.005 and *P* < 0.001, respectively). The concentration of Na^+^ (mmol/L) also differed in calves from V/W embryos vs. calves from fresh and F/T embryos (*P* < 0.05) (140.3 ± 0.6 vs. 139.0 ± 0.6 and 139.0 ± 0.5, respectively) (Figure 1F), and K^+^ (mmol/L) tended to increase in calves when the original embryo was F/T vs. fresh (*P* = 0.089; pdiff = 0.0356). Among the metabolites analyzed (Figure 2), creatinine in calves was clearly affected (*P* = 0.004) by both embryo cryopreservation systems (Figure 2A; F/T: 3.875 ± 0.306 mg/dl and V/W: 3.881 ± 0.326; vs. fresh: 2.770 ± 0.333), as well as by culture medium and calf sex, tending to decrease after colostrum intake (*P* = 0.097; not shown in tables). Urea (Figure 2B) did not differ with embryo cryopreservation but showed a significant dependence on the embryo culture system (*P* = 0.0046). Both hematological parameters (Figure 2), Hb (g/dl) (Figure 2C) and PCV (%) (Figure 2D), differed (*P* < 0.05) between calves born from F/T and V/W embryos, but they did not differ from calves of fresh ones (Hb: 8.565 ± 0.470 vs. 9.844 ± 0.500, and 9.233 ± 0.511; PCV: 25.22 ± 1.38 vs. 28.95 ± 1.47, and 27.12 ± 1.50, respectively). The remainder of Day 0 interactions colostrum^*^embryo cryopreservation did not significantly differ and are shown in Supplementary Table S2.

### Time-course studies in the perinatal period

These studies used *N* = 138 samples, corresponding to Day 0 (*N* = 50), Day 15 (*N* = 44), and Day 30 (*N* = 44). Five calves died before Day 15. On Day 15, two samples were not taken,and, on Day 30, other two samples from different calves were not taken either. Table 1 shows further calf details. We first analyzed the interaction between embryo cryopreservation and calf age at three time points. The sample distribution per groups for F/T, fresh, and V/W resulted in Day 0: 23, 13, and 14; Day 15: 20, 13, and 11; Day 30: 19, 13, and 12, respectively.

Values measured on a temporal scale were mostly independent of embryo cryopreservation and showed changes regarding Day 0 values. However, on Day 30, several parameters were influenced by the previous appearance of diarrhea. Mild diarrhea was either untreated or corrected with diet. Moderate-to-severe diarrhea was treated with diet and nutritional and electrolyte replacement (Calf Lyte Plus, Vetoquinol, Spain), with or without injected sulfadoxinetrimethoprim therapy (Borgal; Virbac, Esplugues de Llobregat, Barcelona, Spain).

**Figure 3:**
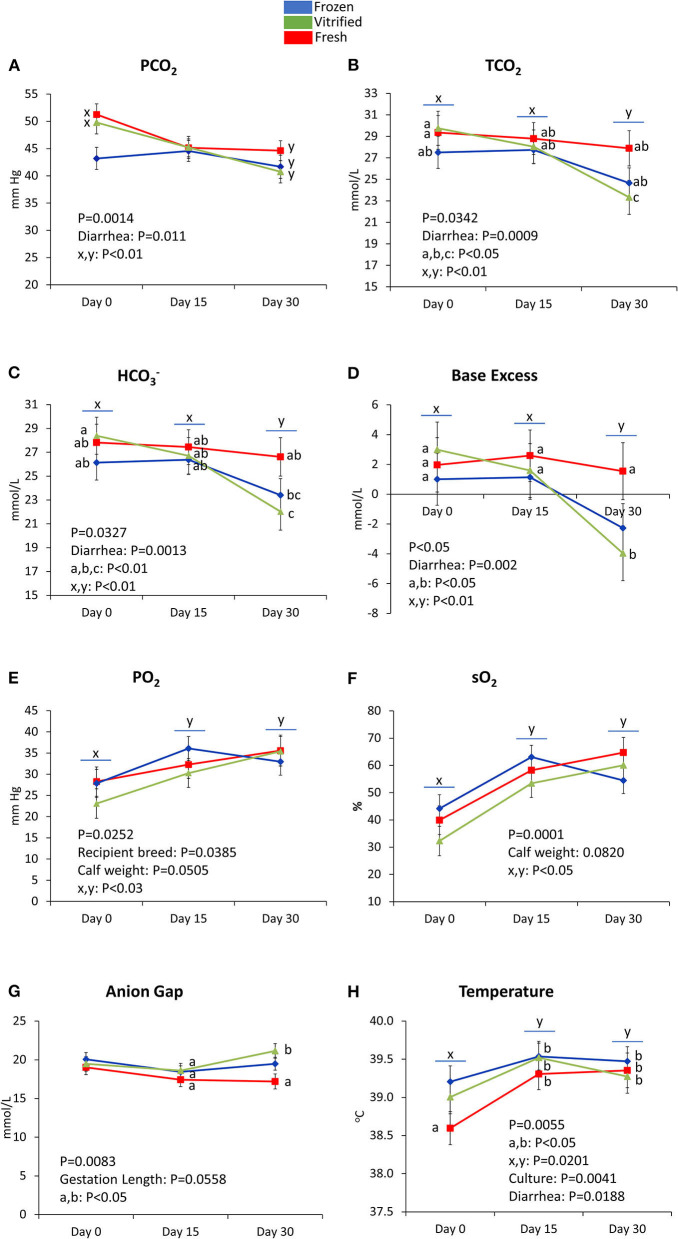
Acid–base equilibrium and blood gas parameters differed on Day 0, Day 15, and/or Day 30 between groups of calves (from frozen/thawed, fresh, and vitrified/warmed original embryos): PCO_2_ **(A)**, TCO_2_ **(B)**, HCO${}_{3}^{-}$**(C)**, base excess **(D)**, PO_2_ **(E)**, sO_2_ **(F)**, anion gap **(G)**, and temperature **(H)**. Data are expressed as LSM ± SEM. Underlined superscripts indicate the day effect, and not-underlined superscripts indicate the interaction between days and cryopreservation.

**Figure 4:**
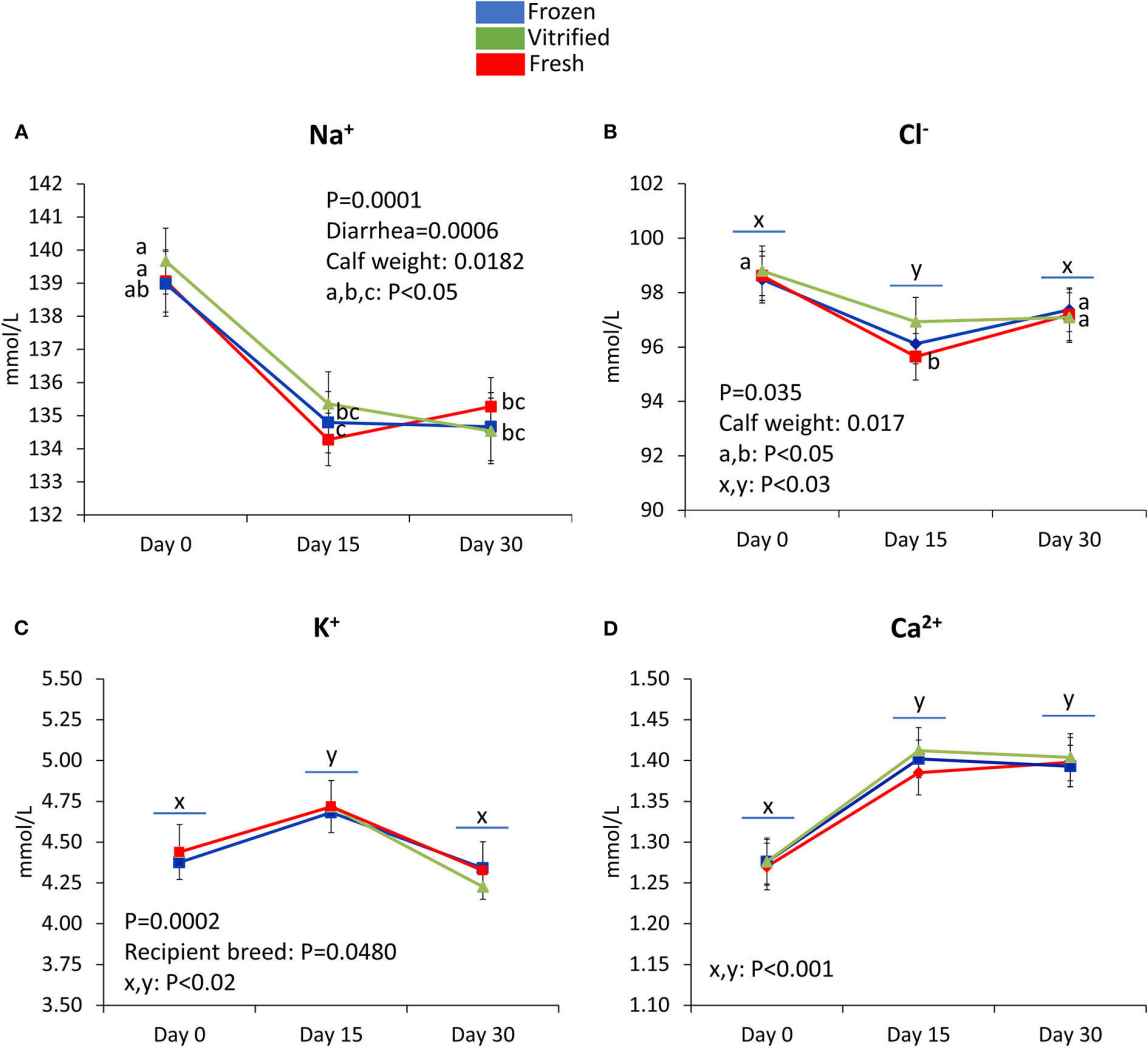
Electrolytes that differ in the three groups of calves in the temporal study (Day 0, Day 15, and Day 30). Na^+^ **(A)**, Cl^−^**(B)**, K^+^ **(C)**, and Ca^2+^ **(D)** concentrations are shown. Data are expressed as LSM ± SEM. Underlined superscripts indicate the day effect, and not-underlined superscripts indicate the interaction between days and the cryopreservation system.

**Figure 5:**
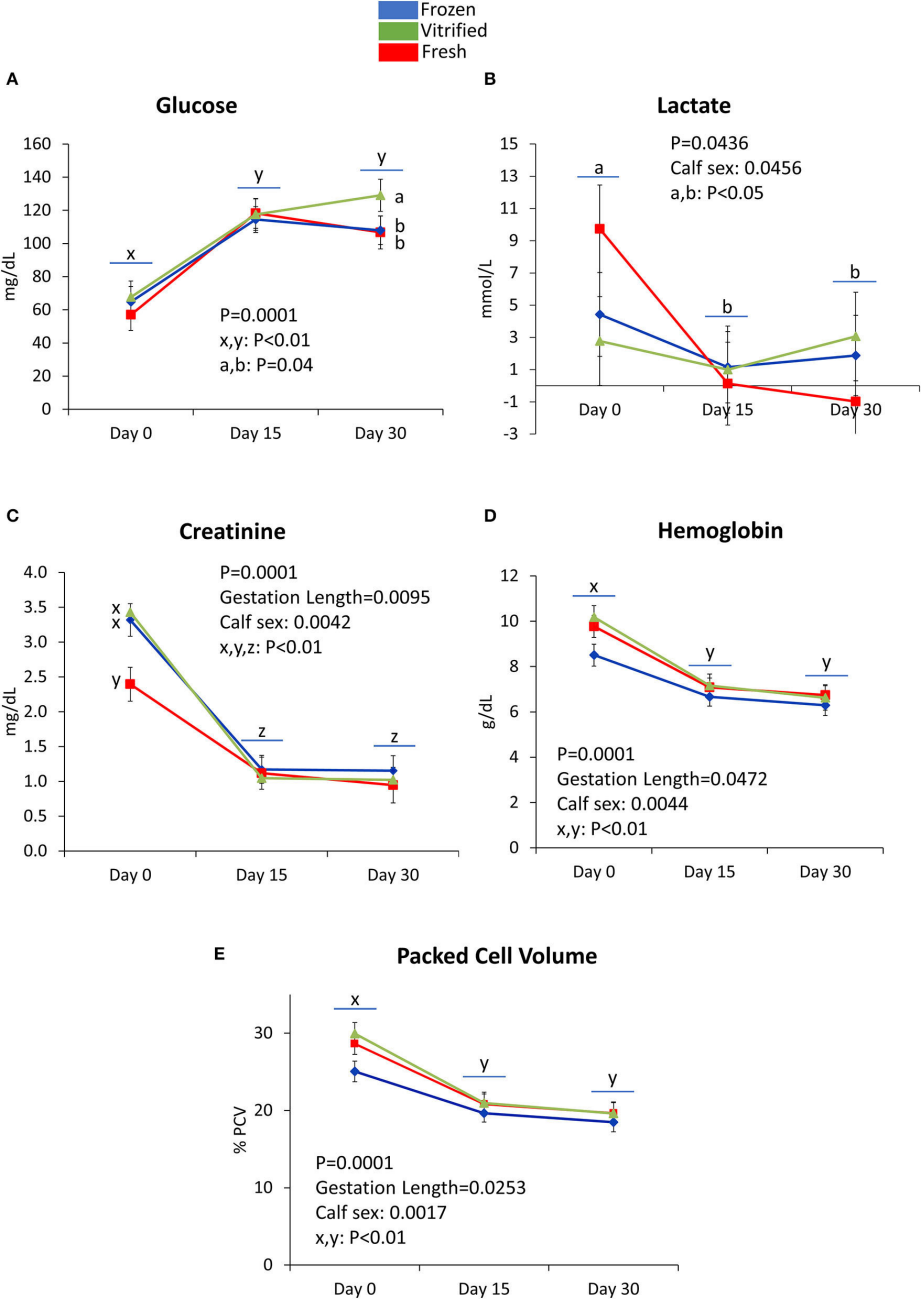
Metabolite concentration [glucose **(A)**, lactate **(B)**, and creatinine **(C)**] and hematological parameters [hemoglobin **(D)** and packed cell volume **(E)**] measured on Day 0, Day 15, and Day 30 differed (*P* < 0.05) between calves derived from frozen/thawed, fresh, and vitrified/warmed embryos. Data are expressed as LSM ± SEM. Underlined superscripts indicate the day effect, and not-underlined superscripts indicate the interaction between days and the cryopreservation system.

Figures 3–5 show values for the day effect and the interaction day^*^cryopreservation. Within acid–base equilibrium and blood gas concentrations parameters (Figure 3), PCO_2_ decreased in calves from fresh and V/W embryos, but remained constant in calves from F/T embryos (Figure 3A), while overall TCO_2_ decreased on Day 30, an effect more marked within calves born after transfer of V/W embryos (Figure 3B) which was also shown by ion bicarbonate (Figure 3C) and BE (Figure 3D). On the contrary, PO_2_ and sO_2_ increased throughout with no incidence of cryopreservation effects (Figures 3E,F respectively). The anion gap also showed an increase in Day 30 over Day 15 values within calves from V/W embryos, while the other time points and groups remained without changes (Figure 3G). Temperature increased in all groups from Day 0 to a plateau on Day 15 and Day 30 (Figure 3H), with a more pronounced, significant rise in calves from fresh embryos. Among calf electrolytes (Figure 4), none was affected by cryopreservation, with Na^+^ decreasing abruptly from Day 0 until Day 15 and Day 30 (Figure 4A), while Cl^−^ showed a transient decrease only on Day 15 (Figure 4B), K^+^ a transient increase on Day 15 (Figure 4C) and Ca^2+^ rose on Day 15 to remain stable up to Day 30 (Figure 4D). Among metabolites analyzed (Figure 5), carbohydrates showed contrary profiles between them, with glucose rising from Day 0 to remain stable on Day 15 and Day 30 (with a significant peak for calves from V/W embryos on Day 30) (Figure 5A), in contrast with lactate, which decreased from Day 0 (Figure 5B). Creatinine showed on Day 0 -with the post-prandial samples—the same changes described above due to embryo cryopreservation, and thereafter decreased to a basal level on Day 15 and Day 30 (Figure 5C). Hematological parameters (Figure 5), Hb (Figure 5D), and PCV (Figure 5E) showed parallel decreases from Day 0 with comparable values between Day 15 and Day 30.

The diarrhea influence on parameters was exerted clearly on Day 30 and particularly in calves from V/W embryos; despite this, such calves did not show a higher incidence of the illness than calves from F/T and fresh embryos (refer to Table 1). Parameters influenced by diarrhea on Day 30 were temperature, PCO_2_, TCO_2_, HCO${}_{3}^{-}$, BE, and Na^+^. Parameters that did not show diarrhea influence on Day 30 were PO_2_, sO_2_, AG, Cl^−^, glucose, lactate, creatinine, PCV, and Hb. Culture conditions (i.e., the presence of 0.1% FCS in culture prior to Day 6) affected calf temperature (not shown in figures), while lactate, creatinine, PCV, and Hb were significantly different between male and female calves. Calf weight (repeatedly measured on days of sampling) affected concentrations of PO_2_, sO_2_, Na^+^, and Cl^−^. Gestation length affected AG, creatinine, PCV, and Hb. The remaining parameters measured did not show time-dependent changes or interaction with the embryo cryopreservation origin of the calves; such values are described in Supplementary Table S3.

Creatinine showed low or no correlation with parameters that can be altered by dehydration (i.e., CRT: R = 0.26752, *P* = 0.0371; PCV: R = 0.18547, *P* = 0.1524; Hb: R = 0.1485, *P* = 0.18721) showing only correlations with PCO_2_ (R = −0.30673; *P* = 0.0241), GL (R = 0.37304; *P* = 0.0031), and chest perimeter (R = 0.30860; *P* = 0.0155) but no correlation with calf weight and size at birth; nor did any other parameter measured. This indicates that cryopreservation accounted for most of the creatinine variation.

A comparison between the values of parameters obtained in our study on Day 0 (before and after colostrum intake), Day 15, and Day 30 and reference values reported in other studies is shown in Supplementary Table S4. Total consistency of our values with a given list of reference values was not observed; in addition, no reference value list showed complete consistency with each other. However, all our specific parameters showed adjustment to at least one reference value at any time, with the exception of urea (lower concentration in our study) and TCO_2_ (which was not measured in the referred studies).

## Discussion

We evaluated calf fitness on Day 0 (before and after colostrum intake) and on Days 15 and 30 after birth to monitor adaptive changes. Part of the parameters measured on Day 30, but not before, was significantly affected by the appearance of diarrhea, which was of mild-to-moderate intensity in most cases, including two deaths. Although the focus of our study was investigating the effects of embryo cryopreservation among IVP calves, we compared our results with recommended values (Cornell University), and values obtained in similar studies (40, 41, 46, 53–58). Overall, parameters measured in our study fitted well with parameters given by other authors in an independent way, but reliable and linear comparisons must be cautiously interpreted because the age and conditions of animals in literature were not always the same as our sampling times, and studies were generally conducted under different nutritional and management diets. Therefore, accounting for the effects of other environmental variables (farm, feeding time and composition, type of food, reproductive methods, such as AI, cloning, other *in vitro* production systems, analytical platforms, and undetermined residual factors) is not feasible (46). The analyzer we used has been positively evaluated in calves (40, 59–61), and although some minor biases were noted between our and other analyzers, they did not interfere with the clinical value of data (62).

### Embryo technologies, calf performance, and growth

*In vitro*-produced calves often come from a prolonged gestation (40, 63), which contributes to increased BW and puts mother and calf at risk and may include a proportion of calves with LOS/AOS phenotype [reviewed by Rivera et al. (64)]. However, in our experimental herd, there were no BW differences between fresh, F/T, and V/W calves. Beef recipients, like AV, have shorter GL than dairy cattle (65) and lower milk yield. For this reason, the recipient breed (Holstein, AV, crossbred) was a factor to correct in our study, which affected nasal flux, conjunctival appearance, Na^+^, and Cl^−^ values at birth. Instead, in our time-course study, the recipient breed only affected PO_2_ and K^+^. Although the transfer of V/W embryos leads to reduced pregnancy and birth rates and increased GL (14), embryo cryopreservation as a source of alteration in newborn calves has been not studied in depth. This is in contrast with data obtained from technologies of embryo production (12, 15). For example, PCV, Hb, urea, and creatinine differ between clones and calves from AI (41), and in our study with cryopreservation. Clones and calves showing AOS present hypoglycemia (41), contrary to our study, where neither glucose nor lactate at birth was affected by cryopreservation. Glucose did not increase after colostrum intake, in accordance with (66), but contrary to Guo and Tao (52), and increased on Day 15, as shown by others (66–68). This is opposed to lactate, which decreases with age (52, 66, 67). However, we do not know the reason for higher glucose in calves from V/W embryos on Day 30, as an isolated finding, although temporary glucose increases have been reported in calves obtained from IVP embryos vs. AI (39). Alterations induced in calves by reproductive techniques may help to identify parameters “sensitive” to injuries. Thus, the type and sense of changes reported between calves from IVP and/or cloned embryos vs. calves from AI could have parallel effects due to cryopreservation in our IVP embryos.

### Acid–base equilibrium and blood gases

The combination of metabolic and respiratory acidosis, due to altered gas exchange between the fetus and the mother, is a major cause of perinatal calf mortality (69, 70). Deviation in acid–base balance entailed lower values of hematic HCO${}_{3}^{-}$, pH, and BE in heavier calves compared with those with more reduced BW (71). In our study, calves from fresh, V/W, and F/T embryos did not differ in BW and GL. Calf overgrowth leads to labor difficulties, which affect at least pH, lactate, and PCO_2_ at birth (72). Gas values, pH, lactate, and BE are early predictors of respiratory compromise and their values correlate with blood levels of lung injury proteins (73). However, in our study, parameters involved in the acid–base equilibrium on Day 0 reflected only slight differences between cryopreserved and fresh embryos, without pathological damage.

Reduced PCO_2_ concentration in F/T calves suggests primary respiratory alkalosis due to hypoxia, pointing to hyperventilation during labor as a probable cause. After colostrum intake, PCO_2_ decreased in F/T and fresh calves but not within V/W. All differences in PCO_2_ disappeared on Day 15 and Day 30, with a PCO_2_ reduction over the days. In contrast, the concentration of HCO${}_{3}^{-}$ was steady on Day 0 and Day 15, being reduced by diarrhea on Day 30. Such a decrease in HCO${}_{3}^{-}$ was more pronounced in V/W calves, perhaps suggesting a higher sensitivity to diarrhea and/or more compromised immune status, which should be investigated in future studies. TCO_2_ and BE, not affected by cryopreservation at birth, were however affected by diarrhea, and in calves from V/W embryos on Day 30. AG, an indicator of diarrhea and metabolic acidosis, showed a slight increase on Day 30 in calves from V/W vs. fresh embryos, which is also consistent with the timing for diarrhea. Interestingly, calves from F/T embryos showed intermediate values between calves from V/W and fresh embryos on Day 30 in TCO_2_, HCO${}_{3}^{-}$, BE, and AG, reflecting that the possible metabolic alteration by diarrhea in F/T did not occur to the greater extent showed by V/W.

Cryopreservation had a limited or no effect on PO_2_ and sO_2_, parameters associated with oxidative stress. Interestingly, sO_2_ does not account for fetal Hb or dysfunctional Hbs, by which sO_2_ is seen here as a measure of the temporal replacement of fetal calf Hb by adult Hb, a process that ends at 13 weeks of age (74, 75). Embryo cryopreservation did not interfere with this normal adaptation to adult life. Consistent with sO_2_, PCV values decreased throughout, reflecting the physiological replacement of large fetal erythrocytes by the lower volume adult cells (76). At the end of pregnancy, the fetus responds to hypoxia with a transient higher concentration of Hb and PCV. Thus, colostrum intake, a rapid erythrocyte replacement, and a decrease in fetal Hb trigger changes in hematological parameters in the first weeks after birth (77–79). Colostrum had the expected restorative effects on calf physiology, and there were no major significant interactions between colostrum and the cryopreserved or fresh status of the original embryos.

Oxidative stress is prevalent in calves from IVP and cloned embryos, which show higher concentrations of free radicals and less counteracting glutathione in the blood than in adult cattle (80, 81). Such higher levels of free radicals were attributed to the onset of breathing after birth, which enhances the contact between lung epithelia and oxygen (80). However, we identified lower PO_2_ and sO_2_ at birth than later on in our three types of calves, and oxygen-based parameters did not vary with embryo cryopreservation or any other effect at birth. We suggest that non-respiratory factors also underlie increased ROS levels in newborn calves.

Measures of acid–base balance (pH, PCO_2_, PO_2_, BE, and HCO${}_{3}^{-}$), PCV, Hb concentration, and blood cells did not differ between calves born from IVP embryos and AI aged 1 and 7 days (40). However, Sangild et al. (68) observed calves from IVP embryos with higher pH, Hb, oxygen contents, and temperature. Electrolytes can in turn increase in calves from IVP embryos at birth [K^+^: (68)] and on Day 7 [K^+^ and Na^+^: (40)] or decrease at birth [K^+^: (40); Na^+^ and Cl^−^: (68)]. We noted a tendency toward elevated K^+^ in calves from fresh over F/T embryos, and we agree with other authors regarding the decrease in Na^+^ and Cl^−^ over time as explained by colostrum and water intake leading to hemodilution (40). In parallel, the Ca^2+^ supply in milk would explain its rise until Day 15, as observed by Sangild et al. (68) although not seen by Rerat et al. (40). Embryo cryopreservation seemed to impose more changes on calves than reported for IVP vs. AI.

Calf temperature at birth did not vary with cryopreservation and decreased with colostrum intake. Nor temperature at birth does differ between calves from IVP and AI calves, despite the fact that the regulating plasmatic 3,5,3′-triiodothyronine (T3) and thyroxine (T4) hormones at birth are lower in calves derived from IVP embryos (40). However, cloned calves show higher temperatures than AI calves until 50 days of age (41) and the expression of genes related to thermogenesis differs in the hypothalamus of young male calves obtained from IVP vs. MOET embryos (38). Collectively, the above suggests that temperature regulatory mechanisms are not specific targets for embryo cryopreservation.

### Protein metabolism: Urea and creatinine

Creatinine concentration was higher at birth in calves from V/W and F/T vs. fresh embryos, indicating a consistent alteration induced by cryopreservation, whatever the cryopreservation technique used. Creatinine also increased in umbilical cord plasma (82) and calf venous blood (39) of IVP vs. AI fetuses. Subsequently, creatinine decreases (78, 83–85) as found in our study. The endogenous metabolism in muscles generates creatinine as waste, in a direct proportion of muscle mass, and blood creatinine concentration does not depend on nutrition. The responsiveness of our calves to the colostrum intake by decreasing creatinine, and the disappearance of differences observed shortly after, indicate reversibility and no obvious damage induced by cryopreservation. The creatinine alteration is in contrast with the steady concentrations of urea among groups on Day 0. Urea concentration depends on nutrition, and its increase in blood indicates protein catabolism (83), although we did not observe any decrease in urea concentration with time. In the ewe, high urea levels in the uterus and oviduct reduce embryo development rates and enhance fetal growth (86, 87), as occurs in culture with serum (88), and our study.

Sex affected Day 0 concentrations of Na^+^ and Cl^−^ (increased in men), but such differences disappeared afterward. However, sex differences in creatinine, PCV, and Hb observed on Day 0 remained throughout. Creatinine concentration was higher in males at birth (40), which can be explained by the larger muscle mass in these calves (40, 89). Our results are consistent with observations of Dillane et al. (46) for Cl^−^, PCV, and pH (which showed a tendency), are contrary for Na^+^, and are not coincident for glucose, HCO${}_{3}^{-}$, PCO_2_, AG, and K^+^ (not affected by sex in our study). Other studies did not find differences attributable to calf sex for the above and other parameters investigated (76, 90).

### Adaptation to extrauterine life

As reported by Schäff et al. (67), we observed how parameters measured in the first month of an age change to adapt to mature life. CRT is a measure of mild-to-moderate dehydration in calves (91). In our story, calves from F/T and V/W embryos seemed to show this moderate dehydration, as their CRT surpassed 3 but not 4 s both before and after colostrum intake. CRT ≥ 3 s reflects a 4.3% reduction in hydration in dairy calves compared with lower refill times (91). PCV is also typically elevated in dehydration (92), as shown in calves from V/W embryos compared with calves from F/T embryos but not within calves from not cryopreserved embryos. Rectal temperature and respiration rates decrease by dehydration (91), but such parameters did not change between calf types. Collectively, V/W embryos led to calves who showed more dehydration signs than calves from F/T embryos, although without reaching a clinical compromise, since CRT cannot be measured in calves with severe dehydration (91, 93). Thus, the pH values of all calf types at birth were higher than 7.2, a clinical limit to judge a calf as acidotic (93), and all types responded to colostrum intake with pH rises between 7.30 and 7.35. Animals over 7 days old should reflect values > 7.31 (94) or > 7.36 (90).

Metabolic adaptation in the calf starts during the third week of life when newly synthesized, calf proteins must replace colostrum-provided and *in utero*-synthesized proteins (83). Disrupting neonatal metabolic adaptation would make the calf susceptible to infectious- and non-infectious diseases. We observed here that calves from V/W embryos showed metabolic traits compatible with diarrhea despite not being clinically affected and/or no more affected than their counterparts.

With the few exceptions marked on Day 0, generally, colostrum intake and subsequent development led to similar calf adaptation of fresh, V/W, and F/T embryos until Day 15. The incidence of diarrhea led to differences on Day 30 within calves from V/W embryos (i.e., two cases treated and two cases not treated within 13 surviving calves). HCO${}_{3}^{-}$, AG, BE, and TCO_2_, differed on Day 30 between calves from V/W vs. fresh and F/T embryos, consistent with significant effects of diarrhea. Other parameters were affected by diarrhea (i.e., temperature, PCO_2_, and Na^+^) but without particular differences between calf groups.

## Conclusion

In the present study, apparently normal calves from cryopreserved embryos show particular clinical and biochemical traits, more pronounced on Day 0 than afterward, as observed with CRT, creatinine, or heartbeat rate. Furthermore, V/W and F/T embryos also led to specific effects on calves, as occurred with PCO_2_ or Na^+^. However, differences from embryo cryopreservation disappeared in calves on Day 15 and Day 30. In contrast, on Day 30, diarrhea altered several parameters, and our results point to V/W embryos as making calves more susceptible to these effects, although overall mortality rates did not differ. The small concentration of serum we used in embryo culture was reflected in the protein metabolism (i.e., urea and creatinine) of calves, although no conclusive statement can be drawn without studying such changes at the cellular level. Anyhow, such differences observed on Day 0 disappeared over time, with the only temperature remaining affected. Colostrum was restorative in the three groups of calves, which indicates an initial similar adaptation to extrauterine life between calves from fresh and cryopreserved embryos. This is the first study to compare the clinical status of calves born from fresh vs. frozen and vitrified IVP embryos. The molecular basis of the observed differences, and whether they persist in progeny as subtle effects not approached herein, requires further investigation.

## Data availability statement

The raw data supporting the conclusions of this article will be made available by the authors, without undue reservation.

## Ethics statement

The animal study was reviewed and approved by the Animal Research Ethics Committees of SERIDA and University of Oviedo (PROAE 33/2020; Resolución de 13 de Noviembre de la Consejería de Medio Rural y Recursos Naturales), in accordance with European Community Directive 86/609/EC.

## Author contributions

EG and JB contributed to the conceptualization and methodology. EG and IG contributed to data curation, software, formal analysis, and writing of the original draft. EG contributed to the funding acquisition and supervision. SC, DM-G, AM, JP-J, and IG contributed to the investigation and contributed to resources. EG, IG, SC, and DM-G contributed to the visualization. EG, IG, AM, JB, and JP-J contributed to the writing of the review and editing. All authors have read and agreed to the published version of the manuscript.

## Funding

This project was partially funded by the European Commission's Horizon 2020 Research and Innovation Program *via* the GLOMICAVE project under grant agreement no. 952908. IG was supported by Ayuda BES-2017-082200 financed by MCIN/AEI/10.13039/501100011033 and FSE “El FSE invierte en tu futuro”. Fondo Europeo de Desarrollo Regional (FEDER).

## Conflict of interest

The authors declare that the research was conducted in the absence of any commercial or financial relationships that could be construed as a potential conflict of interest.

## Publisher's note

All claims expressed in this article are solely those of the authors and do not necessarily represent those of their affiliated organizations, or those of the publisher, the editors and the reviewers. Any product that may be evaluated in this article, or claim that may be made by its manufacturer, is not guaranteed or endorsed by the publisher.
