# Supplementary material for: Fitness of calves born from in vitro-produced fresh and cryopreserved embryos
Source: Front Vet Sci. 2022 Nov 24;9:1006995. doi: 10.3389/fvets.2022.1006995 (PMC9730881; doi:10.3389/fvets.2022.1006995)
Supplement: Supplementary file 1 [file Table_1.docx]

**Supplementary Table S1**

Timeframe validation of whole blood analytes with CG4+ and CHEM8+ cartridges (iSTAT).

Blood samples were taken from jugular vein in heparin-lithium vacuum tubes which were gently shaken 10 times just after collection. Samples were collected from 15-day-old calves (2 males, 2 females) born after transfer of in vitro produced embryos fertilized with frozen/thawed semen from a single bull. The four samples from each series and calf were steadily collected and analyzed in four subsequent periods with the cartridges CG4+ and CHEM8+ (1 blood tube per calf, time and cartridge). The sequence of events was as follows: 1/collection of blood sample; 2/ cartridge CG4+ analysis; 3/ Collection of blood sample; 4/ Cartridge CHEM8+ analysis. The iSTAT instrument was kept at constant temperature in a controlled facility in the experimental farm besides the calves’ barn.

**Supplementary Table S1A**

Parameters analyzed in calf blood (CG4+ cartridge)

| Reading Time | | |  |  |  |  |  |  |  |  |
| --- | --- | --- | --- | --- | --- | --- | --- | --- | --- | --- |
| Period | Min | Interval | pH | pCO_2_ | pO_2_ | Base Excess | HCO_3_ | TCO_2_ | sO_2_ | Lactate |
| 1 | 3.25 | 3-4 | 7.417 | 42.85 | 33.00 | 3.250 | 27.92 | 29.25 | 62.00 | 1.745x |
| 2 | 7.00 | 6-8 | 7.416 | 42.97 | 33.50 | 3.500 | 27.95 | 29.25 | 62.50 | 1.777x |
| 3 | 10.75 | 10-11 | 7.395 | 45.45 | 33.75 | 3.000 | 28.05 | 29.25 | 61.75 | 1.782x |
| 4 | 14.75 | 13-16 | 7.401 | 45.05 | 33.25 | 3.500 | 28.25 | 29.75 | 57.00 | 1.980y |
|  |  |  |  |  |  |  |  |  |  |  |
| ±SE | 0.42 |  | 0.007 | 1.24 | 2.48 | 0.390 | 0.38 | 0.54 | 3.53 | 0.03 |
| P value | <0.001 |  | 0.158 | 0.364 | 0.997 | 0.776 | 0.927 | 0.887 | 0.676 | 0.001 |

**Supplementary Table S1B**

Parameters analyzed in calf blood (CHEM8+ cartridge)

| Reading Time | | |  |  |  |  |  |  |  |  |  |  |
| --- | --- | --- | --- | --- | --- | --- | --- | --- | --- | --- | --- | --- |
| Period | Min | Interval | Na^+^ | K^+^ | Cl^-^ | Ca^2+^ | Glucose | Urea | Creatinine | PCV | Hemoglobin | Aniongap |
| 1 | 2.7 | 2-4 | 137.7 | 4.750 | 96.00 | 1.365 | 123.5 | 4.250 | 1.250 | 23.50 | 7.975 | 21.00 |
| 2 | 6.2 | 5-7 | 137.2 | 4.825 | 97.00 | 1.367 | 123.5 | 4.250 | 1.275 | 24.25 | 8.250 | 19.25 |
| 3 | 9.2 | 8-10 | 137.5 | 4.725 | 96.50 | 1.372 | 123.2 | 4.500 | 1.250 | 24.25 | 8.225 | 19.75 |
| 4 | 13.2 | 12-14 | 137.5 | 4.850 | 96.25 | 1.380 | 124.2 | 4.500 | 1.225 | 24.75 | 8.400 | 20.25 |
|  |  |  |  |  |  |  |  |  |  |  |  |  |
| ±SE | 0.4 |  | 0.2 | 0.067 | 0.41 | 0.007 | 0.4 | 0.144 | 0.029 | 0.37 | 0.129 | 0.70 |
| P value | <0.001 |  | 0.280 | 0.532 | 0.405 | 0.535 | 0.608 | 0.436 | 0.691 | 0.202 | 0.208 | 0.384 |
