# Supplementary material for: Fitness of calves born from in vitro-produced fresh and cryopreserved embryos
Source: Front Vet Sci. 2022 Nov 24;9:1006995. doi: 10.3389/fvets.2022.1006995 (PMC9730881; doi:10.3389/fvets.2022.1006995)
Supplement: Supplementary file 2 [file Table_2.docx]

**Supplementary Table S2**

Day-0 parameters in calves whose values did not differ in accordance with the cognate embryo cryopreservation

|  | Frozen | Fresh | Vitrified | Units | P value |
| --- | --- | --- | --- | --- | --- |
| Temperature | 39.08±0.20 | 38.67±0.22 | 39.12±0.21 | ^○^C | 0.138 |
| Conjunctival | 1.890±0.041 | 1.930±0.045 | 1.913±0.044 | AU | 0.731 |
| Nasal | 1.787±0.044 | 1.809±0.048 | 1.7770.047 | AU | 0.814 |
| Respiration | 43.07±2.70 | 38.57±2.94 | 40.06±2.88 | breathings/min | 0.369 |
| pH | 7.321±0.019 | 7.301±0.021 | 7.321±0.020 |  | 0.611 |
| HCO_3_^-^ | 25.87±0.94 | 28.22±1.02 | 27.57±1.00 | mmol/L | 0.100 |
| TCO_2_ | 27.47±0.94 | 29.89±1.03 | 29.10±1.01 | mmol/L | 0.098 |
| Lactate | 6.783±2.543 | 8.540±2.767 | 4.479±2.706 | mmol/L | 0.362 |
| K^+^ | 4.323±0.115 | 4.624±0.125 | 4.526±0.122 | mmol/L | 0.089 |
| Cl^-^ | 97.77±0.75 | 97.60±0.81 | 98.73±0.80 | mmol/L | 0.320 |
| Ca^2+^ | 1.304±0.023 | 1.314±0.025 | 1.304±0.024 | mmol/L | 0.924 |
| Glucose | 79.19±8.57 | 78.25±9.33 | 80.07±9.12 | mg/dL | 0.982 |
| Urea | 5.311±1.401 | 3.581±1.524 | 5.365±1.490 | mg/dL | 0.492 |
| Anion Gap | 20.73±0.62 | 19.10±0.67 | 19.90±0.66 | mmol/L | 0.104 |
| PO_2_ | 25.72±2.65 | 29.18±2.93 | 25.44±2.82 | mm Hg | 0.438 |
| sO_2_ | 39.81±4.52 | 39.88±4.50 | 35.88±4.81 | % | 0.637 |

AU: Arbitrary units
