# Supplementary material for: Fitness of calves born from in vitro-produced fresh and cryopreserved embryos
Source: Front Vet Sci. 2022 Nov 24;9:1006995. doi: 10.3389/fvets.2022.1006995 (PMC9730881; doi:10.3389/fvets.2022.1006995)
Supplement: Supplementary file 3 [file Table_3.docx]

**Supplementary Table S3**

Time course of variable values that did not differ between sampling days (not shown in figures).

| Parameter | Day-0 | Day-15 | Day-30 | P value |
| --- | --- | --- | --- | --- |
| CRT | 3.136±0.215 | 3.056±0.178 | 2.999±0.205 | 0.867 |
| Conjunctival | 1.923±0.049 | 1.978±0.040 | 1.960±0.046 | 0.494 |
| Nasal | 1.873±0.055 | 1.868±0.046 | 1.849±0.053 | 0.911 |
| Heartbeats | 141.7±5.8 | 138.4±4.8 | 137.6±5.6 | 0.812 |
| Respiration | 40.78±2.29 | 41.77±1.89 | 38.73±2.19 | 0.282 |
| Urea | 6.497±1.228 | 6.066±1.013 | 5.921±1.170 | 0.917 |
| pH | 7.355±0.024 | 7.365±0.019 | 7.328±0.023 | 0.181 |

Day-0 includes only post-prandial samples.
