# Supplementary figures and images for: Fitness of calves born from in vitro-produced fresh and cryopreserved embryos
Source: Front Vet Sci. 2022 Nov 24;9:1006995. doi: 10.3389/fvets.2022.1006995 (PMC9730881; doi:10.3389/fvets.2022.1006995)

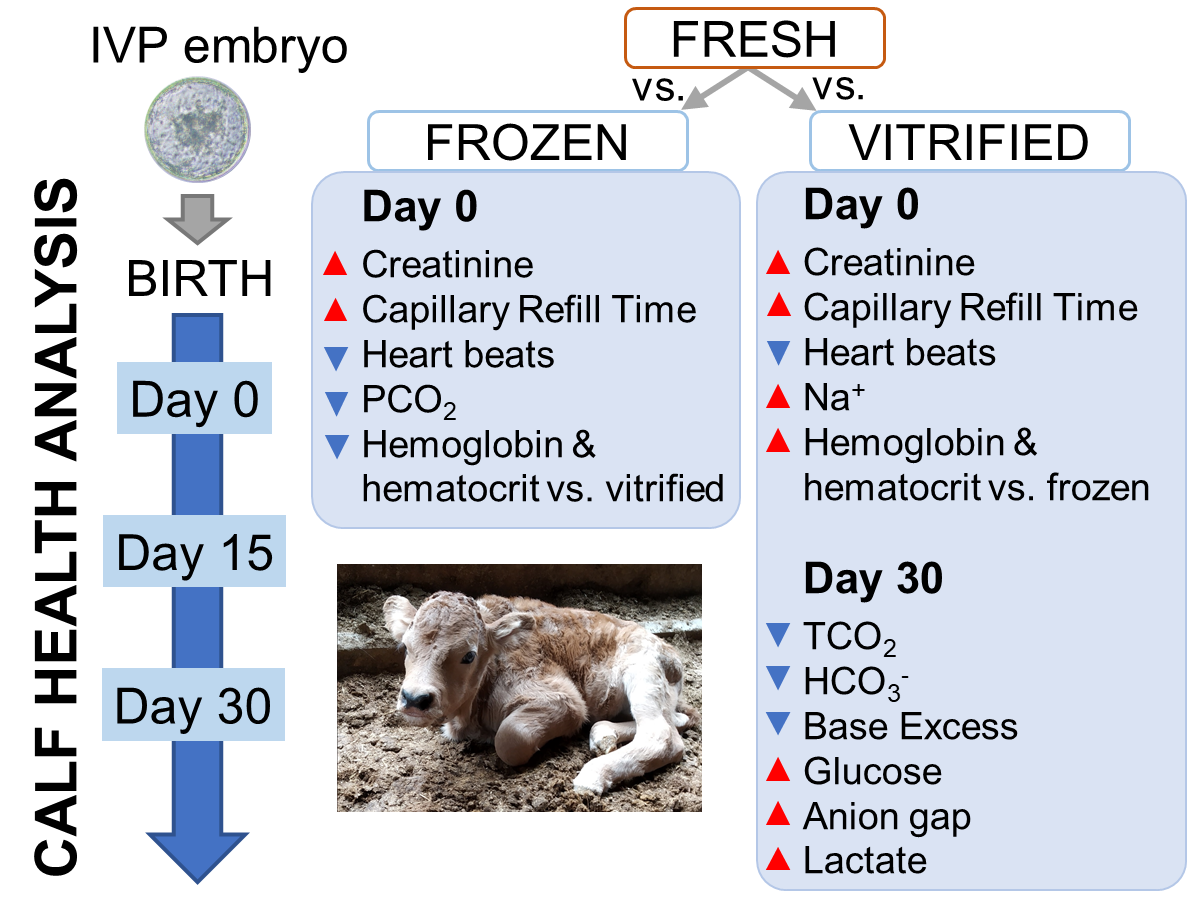

Supplement: Supplementary file 5 [file Image_1.tif]
